# The learning curve of endoscopic endonasal transsphenoidal surgery for pituitary adenomas with different surgical complexity

**DOI:** 10.3389/fsurg.2023.1117766

**Published:** 2023-03-21

**Authors:** Jinxiang Huang, Xinjie Hong, Zheng Cai, Qian Lv, Ying Jiang, Wei Dai, Guohan Hu, Yong Yan, Juxiang Chen, Xuehua Ding

**Affiliations:** ^1^ Department of Neurosurgery, Shanghai Institute of Neurosurgery, Shanghai Changzheng Hospital, Naval Medical University, Shanghai, China; ^2^ Department of Endocrinology, Shanghai Changzheng Hospital, Naval Medical University, Shanghai, China; ^3^ Cerebrovascular Diseases Center, Department of Neurosurgery, Renji Hospital, Shanghai, China; ^4^ Department of Neurosurgery, Changhai Hospital, Naval Medical University, Shanghai, China

**Keywords:** pituitary adenoma, endoscopic transsphenoidal surgery, outcomes, surgical complexity, learning curve

## Abstract

**Objective:**

To investigate the learning curve under different surgical complexity in endoscopic transsphenoidal approach for pituitary adenoma.

**Methods:**

273 patients undergoing endoscopic transsphenoidal surgery for pituitary adenoma were collected retrospectively and divided into three groups chronologically (early, middle, and late periods). Surgical complexity was differentiated based on Knosp classification (Knsop grade 0–2 vs. Knosp grade 3–4), tumor maximum diameter (MD) (macroadenomas vs. giant adenomas), and history of previous surgery for pituitary adenoma (first operation vs. reoperation). Then the temporal trends in operative time, surgical outcomes, and postoperative complications were evaluated from early to late.

**Results:**

The median operative time decrease from 169 to 147 min across the three periods (*P* = 0.001). A significant decrease in operative time was seen in the simple groups [Knosp grade 0–2 adenoma (169 to 137 min, *P* < 0.001), macroadenoma (166 to 140 min, *P* < 0.001), and first operation (170.5 to 134 min, *P* < 0.001)] but not in their complex counterparts (*P* > 0.05). The GTR rate increased from 51.6% to 69.2% (*P* = 0.04). The surgical period was an independent factor for GTR in the simple groups [Knosp grade 0–2 adenoma: OR 2.076 (95%CI 1.118–3.858, *P* = 0.021); macroadenoma: OR = 2.090 (95%CI 1.287–3.393, *P* = 0.003); first operation: OR = 1.809 (95%CI 1.104–2.966, *P* = 0.019)] but not in the complex groups. The biochemical cure rate increased over periods without statistical significance (from 37.5% to 56.3%, *P* = 0.181). Although intraoperative CSF leakage rose (from 20.9% to 35.2%) and postoperative CSF leakage reduced (from 12.1% to 5.5%), there was no statistically significant trend across the three time periods (*P* > 0.05).

**Conclusion:**

This study showed that complex operations might have a prolonged learning curve. Differentiating surgical difficulty and using multivariate combined analysis may be more helpful in clinical practice.

## Introduction

Pituitary adenomas (PAs) are the third most common intracranial tumors, with an annual incidence rate of 5.8 cases per 100,000 population (1), accounting for 10%–15% of all center nervous system tumors (2). Surgical treatment is a first-line option for pituitary tumors other than PRL-secreting adenomas. The transsphenoidal approach for PA removal has a history of over a century since Schloffer first reported it in 1907 (3). Benefiting from the microscope introduction by Hardy (4), the transsphenoidal approach gradually replaced the traditional transcranial approach as a preferred procedure for most pituitary tumors. After the endoscope was introduced to this field in the early 1990s by Jho and Carrau (5), it has been mastered by increasing neurosurgeons with continuous improvement.

The learning curve is a common issue when transitioning from a microscopic to an endoscopic approach. Various factors, including complex anatomy and a diverse case mix (6), may impact the learning curve. Some prior studies have identified and evaluated the endoscopic transsphenoidal surgery (ETS) learning curve for PA (7, 8). However, only a few have examined the learning curve in various complex situations. Therefore, this paper presented our consecutive series of 273 patients with PA treated with ETS over 7 years. Patients were grouped according to factors that may affect surgical complexity. Then we analyzed the different temporal trends in operative time, extent of resection, biochemical cure (BC), and postoperative complications.

## Materials and methods

### Patient selection and data collection

The study was approved by the Institutional Review Board of Shanghai Changzheng Hospital (No. 2021SLYS6). We retrospectively reviewed adult patients (>18 years) who underwent pure endoscopic endonasal transsphenoidal surgery for pituitary tumors at our department from December 1, 2014, to August 31, 2021. All tumors were confirmed to be PA histologically. Patients receiving stereotactic radiosurgery or radiotherapy for PA before endoscopic surgery were excluded. A multidisciplinary team evaluated, treated, and followed up on all patients. Basic information, radiological and surgical characteristics, and outcomes such as operative time, the extent of resection, BC rate, and postoperative complications were recorded. We divided the patients into three groups in chronological order, each with the same number of cases, defined as the early period group (91 cases), middle period group (91 cases), and late period group (91 cases). Then we investigated the temporal trends in surgical outcomes from the early to the late period to reflect the surgeon's proficiency in ETS as the number of cases accumulated.

### Surgical procedure

All procedures were performed without the help of an ENT surgeon by our senior neurosurgeons, Dr. Xuehua Ding, Dr. Guohan Hu, and Dr. Juxiang Chen. Before using the endoscopic transsphenoidal approach, they all had over ten years of experience in microscopic transnasal transsphenoidal surgery.

The surgical procedures were performed under general anesthesia. The patient was placed in the supine position. We preferred using the right nostril for surgery in patients without a deviated nasal septum. If not, the choice of the nostril for operation depended on the orientation of nasal septum deviation to provide a wider working area. After creating an incision on the septal mucosa, a posterior septectomy and sphenoidotomy were performed. The anterior wall of the sphenoidal sinus was widened with Kerrison rongeur to obtain optimal identification of anatomical landmarks, including the sellar floor, suprasellar notch, clival, bilateral optical, and carotid protuberance. Neuro-navigation and micro-Doppler were used when the tumor infiltrated the skull base. Once the surgical route was confirmed, we drilled a hole in the sellar floor and circumferentially widened it with Kerrison rongeur. In the case of a tumor with a large suprasellar extension, the sellae tuberculum was removed. Sellar dura was incised in an “X” or “Y” fashion. Typically, tumor removal was started inferiorly and laterally to the cavernous sinus's medial wall. The upper part of the tumor was finally removed using curettes and micro spatula in a posteroanterior direction until the redundant diaphragma sellae descended into the intrasellar space. Each folded area of the diaphragm sellae was explored by various angled curettes. Following tumor removal, a 30° angled endoscope was placed into the sellar to explore for any residual tumor.

Intraoperative cerebral spinal fluid (CSF) leakage was further confirmed by the Valsalva maneuver after intrasellar hemostasis. Ordinarily, the sellar floor was reconstructed without a CSF leak using artificial dura mater and fibrin glue. If an intraoperative CSF leak was encountered, a multilayer reconstruction was performed. Autologous fat was placed into the resection cavity to eliminate dead space. Fascia lata, or artificial dura mater, was used as an inlay graft for dural reconstruction. After sealing with fibrin glue, the skull base defect was covered with a pedicled nasoseptal flap (9). A postoperative lumbar drain was placed for 5–7 days. Nasal packing was used in selected cases.

### Differentiating surgical complexity

Knosp classification, tumor maximum diameter (MD), and history of prior surgery for PA were used to differentiate the surgical complexity. All patients underwent magnetic resonance imaging (MRI) of the sellar region preoperatively and postoperatively, respectively. The MD in anterioposterior, cephalocaudal, and transverse adenomas was measured. MD classified PAs as microadenoma (MD < 1 cm), macroadenoma (1 cm ≤ MD < 4 cm), and giant adenoma (MD ≥ 4 cm). According to the modified Knosp classification (10), the patients were divided into the Knosp grade 0–2 group and the Knosp grade 3–4 group. Knosp grade 3 or 4 adenomas were considered as adenomas with cavernous sinus invasion (11). The first operation was defined as surgery for patients who had no history of prior surgery for pituitary adenomas, while reoperation was defined as surgery for patients who had already undergone one or more pituitary adenomectomy.

Compared with surgery for Knosp grade 0–2 adenoma, macroadenoma, and first operation, surgery for Knosp grade 3–4 adenoma, giant adenoma, and reoperation were defined as relatively complex operations. Microadenomas were excluded when analyzing the effect of different maximum tumor diameters on the learning curve because there were too few (6 cases).

### Surgical outcome assessment

Postoperative MRI was evaluated to determine whether total tumor resection was achieved. For non-functioning pituitary adenoma (NFPA), gross total resection (GTR) was defined as the absence of the adenoma on postoperative MRI, while for functioning pituitary adenoma (FPA), total resection on imaging along with BC was considered as achieving a GTR.

Baseline hormone level was tested preoperatively, and then endocrinological work-up was repeated at 1 day, 7 days, 3 months, 6 months, and then annually after that. Perioperative hormonal status was evaluated by endocrinologists of our multidisciplinary team. For growth hormone (GH)-secreting adenoma, BC was determined by random GH < 2.5 ng/ml with normalized sex- and age-adjusted IGF-1 level or a nadir GH < 1 ng/ml after oral glucose tolerance test. For prolactin (PRL)-secreting adenoma, BC was determined by serum PRL level <20 ng/ml in men or <25 ng/ml in women. For adrenocorticotrophin (ACTH)-secreting adenomas, BC was determined by a morning serum cortisol value <5 μg/dl or urinary free cortisol level <10–20 μg/d within 7 days after surgery.

### Statistical analysis

While variables with a normal distribution were described as mean and standard deviation, variables without a normal distribution were described as median and range. The categorical variables were presented as frequency and percentage. One-way ANOVA or Kruskal-Wallis test was used to compare the differences between the three periods. When appropriate, categorical data were compared using the Chi-square test or Fisher's exact test. Binary logistic regression was conducted to identify independent risk factors related to GTR. A *P*-value < 0.05 was considered statistically significant. Data were analyzed using SPSS version 26 (IBM Corp., Armonk, NY, USA).

## Results

### Patient demographics

Our study included 273 consecutive cases, and the patient characteristics are summarized in Table 1. Of these patients, 137 (50.2%) were male, and 136 (49.8%) were female. The mean age of patients at the time of presentation was 52.42 ± 13.37 years, and the average body mass index (BMI) was 24.85 ± 3.34 kg/m^2^. Visual deficits (124 cases, 45.4%) and headache (89 cases, 32.6%) were the most common symptoms, followed by dizziness (71 cases, 26.0%), cranial nerve paralysis (15 cases, 5.5%) and symptoms caused by abnormal hormone secretion including galactorrhea-amenorrhea syndrome in 25 women, hyposexuality in 12 men, and typical facial feature of acromegaly in 22 patients. No significant difference was seen in sex (*P* = 0.649), age (*P* = 0.194), and BMI (*P* = 0.998) between the three periods.

**Table 1:** Preoperative patient and tumor characteristics.

Patient & tumor characteristics	Early period (*n* = 91)	Middle period (*n* = 91)	Late period (*n* = 91)	*P* value
Sex, *n* (%)				0.649
Male	47 (51.6)	42 (46.2)	48 (52.7)	
Female	44 (48.4)	49 (53.8)	43 (47.3)	
Age, mean (SD), y	50.56 (14.22)	52.55 (12.85)	54.14 (12.90)	0.194
Body mass index (BMI), mean (SD), kg/m^2^	24.86 (3.56)	24.84 (3.44)	24.83 (3.04)	0.998
Preoperative visual defect, *n* (%)	43 (47.3)	35 (38.5)	46 (50.5)	0.239
Preoperative headache, *n* (%)	29 (31.9)	32 (35.2)	28 (30.8)	0.805
Tumor volume, median (range), cm^3^	5.20 (0.14–126.48)	5.51 (0.17–29.35)	4.56 (0.14–51.30)	0.629
Maximum diameter (MD)				0.491
Microadenomas (MD < 1 cm)	2 (2.2)	3 (3.3)	1 (1.1)	
Macroadenomas (1 ≤ MD < 4 cm)	73 (80.2)	77 (84.6)	81 (89.0)	
Giant adenomas (MD ≥ 4 cm)	16 (17.6)	11 (12.1)	9 (9.9)	
Tumor type, *n* (%)				0.309
NFPA	59 (64.8)	64 (70.3)	59 (64.8)	
GH-secreting	15 (16.5)	20 (22.0)	15 (16.5)	
PRL-secreting	15 (16.5)	7 (7.7)	16 (17.6)	
ACTH-secreting	1 (1.1)	0 (0)	1 (1.1)	
TSH-secreting	1 (1.1)	0 (0)	0 (0)	
Knosp grade, *n* (%)				0.829
0	7 (7.7)	7 (7.7)	3 (3.3)	
1	18 (19.8)	23 (25.3)	24 (26.4)	
2	30 (33.0)	26 (28.6)	33 (36.3)	
3	25 (27.5)	26 (28.6)	22 (24.2)	
4	11 (12.1)	9 (9.9)	9 (9.9)	
Prior resection, *n* (%)	13 (14.3)	14 (15.4)	14 (15.4)	0.972

SD, standard deviation; NFPA, non-functioning pituitary adenoma; GH, growth hormone; PRL, prolactin; ACTH, adrenocorticotrophin; TSH, thyroid-stimulating hormone.

### Tumor characteristics

The median tumor volume was 5.13 cm^3^ (range, 0.14–126.48 cm^3^, Table 1). No difference in the tumor volume was found between the three periods (*P* = 0.469). In terms of tumor MD, macroadenomas accounted for the majority of cases (231 cases, 84.6%), followed by giant adenomas in 36 cases (13.2%) and microadenomas in only 6 cases (2.2%). The most frequently encountered type of adenoma was NFPA (182 patients, 66.7%), followed by GH-secreting adenoma (50 patients, 18.3%) and PRL-secreting adenoma (38 patients, 13.9%). A similar distribution of tumor MD (*P* = 0.491), tumor type (*P* = 0.309), and Knosp classification (*P* = 0.829) were seen among the three periods. Forty-one patients had undergone previous surgery for PA.

### Operative time

**Figure 1:**
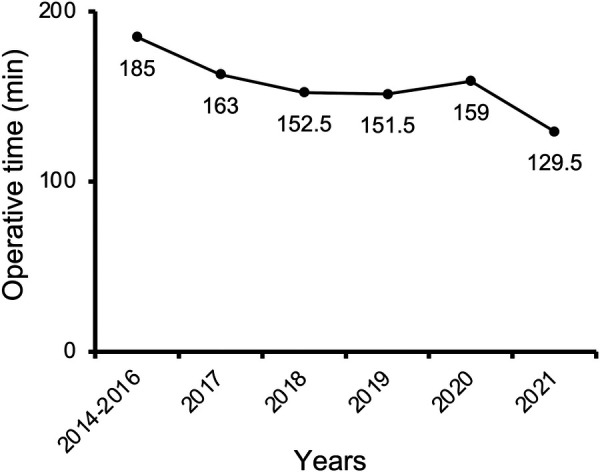
Temporal trend of operative time for endoscopic transsphenoidal surgery over the study period.

The median operative time was 155 min (68–385 min, Table 2). Operative time decreased significantly from 169 min in the early period to 147 min late (*P* = 0.001). We created a year-by-year breakdown in our series to elucidate further the temporal trend in operative time (Figure 1). Thirty-seven ETS were performed during the first three years (2014–2016) with a median operative time of 185 min (102 to 381 min). After that, the median duration of surgery decreased steeply to 163 min (range, 85–385 min) in 2017 and then remained stable for three years [2018: 152.5 min, range (92–288 min); 2019: 151.5 min, range (80–333 min); 2020: 159 min, range (81–325 min)], while another significant decline (18.6%) was seen in the final year [2021: 129.5 min, range (68–250 min)].

**Table 2:** Surgical outcomes and complications in the three periods.

Surgical outcomes & complications	Total	Early period	Middle period	Late period	*P* value
Operative time, median(range), min	155 (68–385)	169 (85–385)	152 (80–333)	147 (68–325)	0.001^*^
Gross total resection (GTR), *n* (%)					0.040^*^
Yes	169 (61.9)	47 (51.6)	59 (64.8)	63 (69.2)	
No	104 (38.1)	44 (48.4)	32 (35.2)	28 (30.8)	
GTR of non-PRL-secreting adenoma, *n* (%)					0.044^*^
Yes	152 (64.7)	42 (55.3)	54 (64.3)	56 (74.7)	
No	83 (35.3)	34 (44.7)	30 (35.7)	19 (25.3)	
Biochemical cure (BC)
Total	46/91 (50.5)	12/32 (37.5)	16/27 (59.3)	18/32 (56.3)	0.181
GH-secreting	27/50 (54.0)	6/15 (40.0)	11/20 (55.0)	10/15 (66.7)	0.340
PRL-secreting	17/38 (44.7)	5/15 (33.3)	5/7 (71.4)	7/16 (43.8)	0.273
Perioperative CSF leakage, *n* (%)
Intraoperative CSF leakage	77 (28.2)	19 (20.9)	26 (28.6)	32 (35.2)	0.101
Postoperative CSF leakage	22 (8.1)	11 (12.1)	6 (6.6)	5 (5.5)	0.216

PRL, prolactin; GH, growth hormone; CSF, cerebral spinal fluid.

^*^ Statistical significance at *p* < .05.

**Figure 2:**
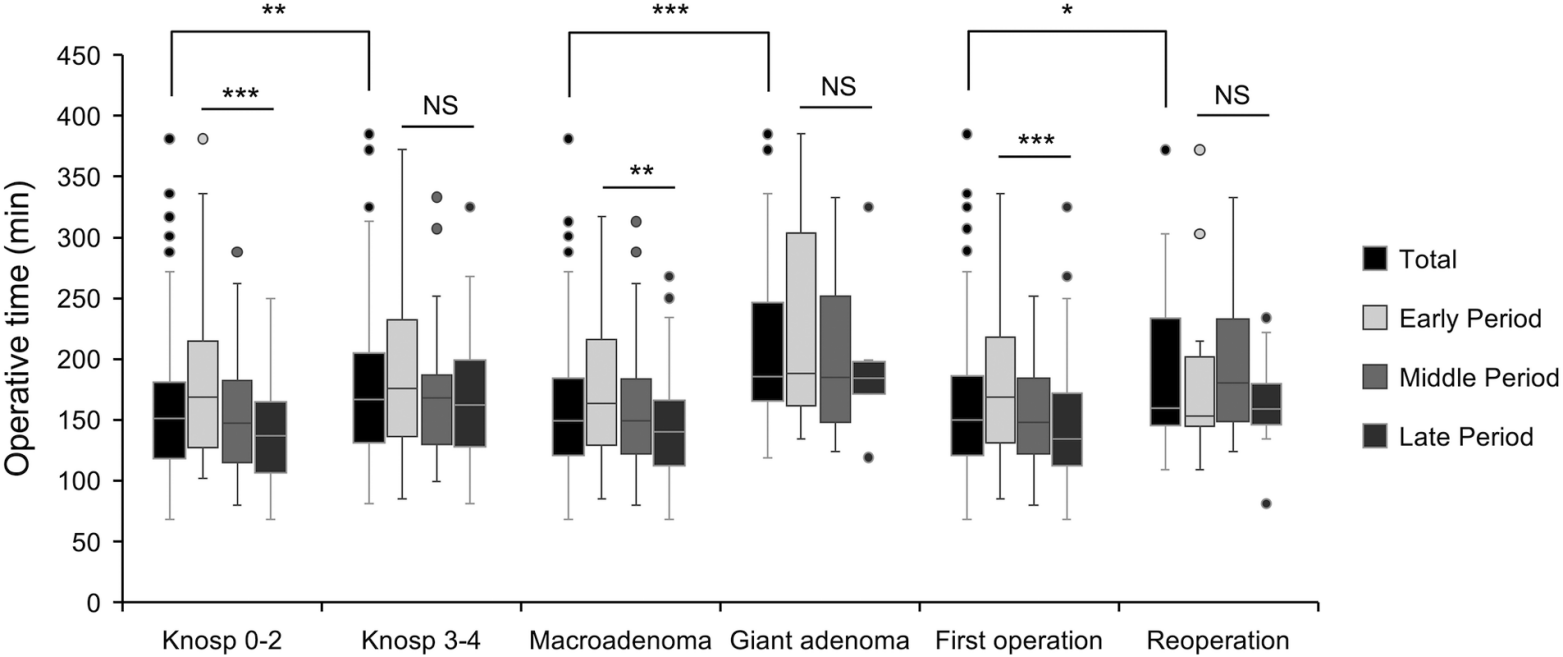
The operation time for pituitary adenomas with different surgical complexity. Comparison of total (black bars), early period (silver bars), middle period (light gray bars), and late period (dark gray bars). Data were compared by Kruskal-Wallis test, * indicates *P* < 0.05, ** indicates *P* < 0.01, *** indicates *P* < 0.001, and NS indicates not significant (*P* > 0.05).

The temporal trends in operative time for each subgroup are described in Figure 2. Overall, the median operative time for complex operations (Knosp grade 3–4 adenomas: 166.5 min; giant adenomas: 185.5 min; reoperations: 166.0 min) was significantly longer than that for their counterpart groups (Knosp grade 0–2 adenomas: 151 min, *P* = 0.002; macroadenomas: 149.0 min, *P* < 0.001; first operations: 151.5 min, *P* = 0.024). For patients with Knosp grade 0–2 adenomas, operative time decreased gradually across periods from 169 min to 147.5 min to 137 min in the late period with a statistical significance (*P* < 0.001). Similar downward trends in operation time were also found in patients with macroadenomas (166.0 min to 149.0 min to 140.0 min, *P* = 0.001) and patients undergoing the first operation (170.5 min to 148.0 min to 134.0 min, *P* < 0.001). Although operation time decreased over time in the giant adenoma group (188.0 min to 185.0 min to 184.0 min, *P* = 0.922) and Knosp grade 3–4 adenoma group (175.5 min to 168.0 min to 162.0 min, *P* = 0.183), no statistical difference was found between the three periods, indicating the decreasing trend was relatively slower. For patients undergoing reoperation, no clear temporal trend in operation time was found (153.0 min to 180.5 min to 159.0 min, *P* = 0.417).

### GTR rate

**Figure 3:**
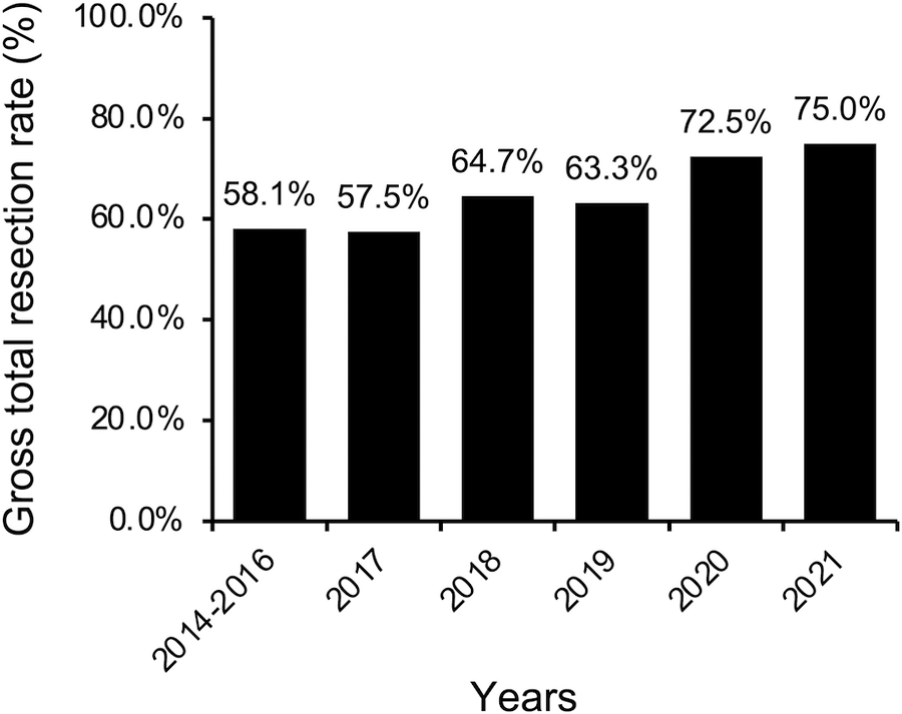
Temporal trend of gross total resection (GTR) rate over the study period.

For the entire cohort, GTR was achieved in 169 cases (61.9%, Table 2). A more conservative surgical strategy was utilized for PRL-secreting adenomas, particularly those invading the cavernous sinus, because surgery followed by an adjuvant dopamine agonist can effectively control most of these adenomas. Thus, PRL-secreting adenomas were excluded from the subsequent analysis of the GTR rate. For non-PRL-secreting adenomas, GTR was achieved in 152 of 235 cases (64.7%). The rate of GTR increased significantly from 55.3% in the early period to 64.3% in the middle period and reached 74.7% in the late period (*P* = 0.044). When broken down by year, the GTR rate increased slowly and steadily from 58.1% to 75.0% (Figure 3).

**Figure 4:**
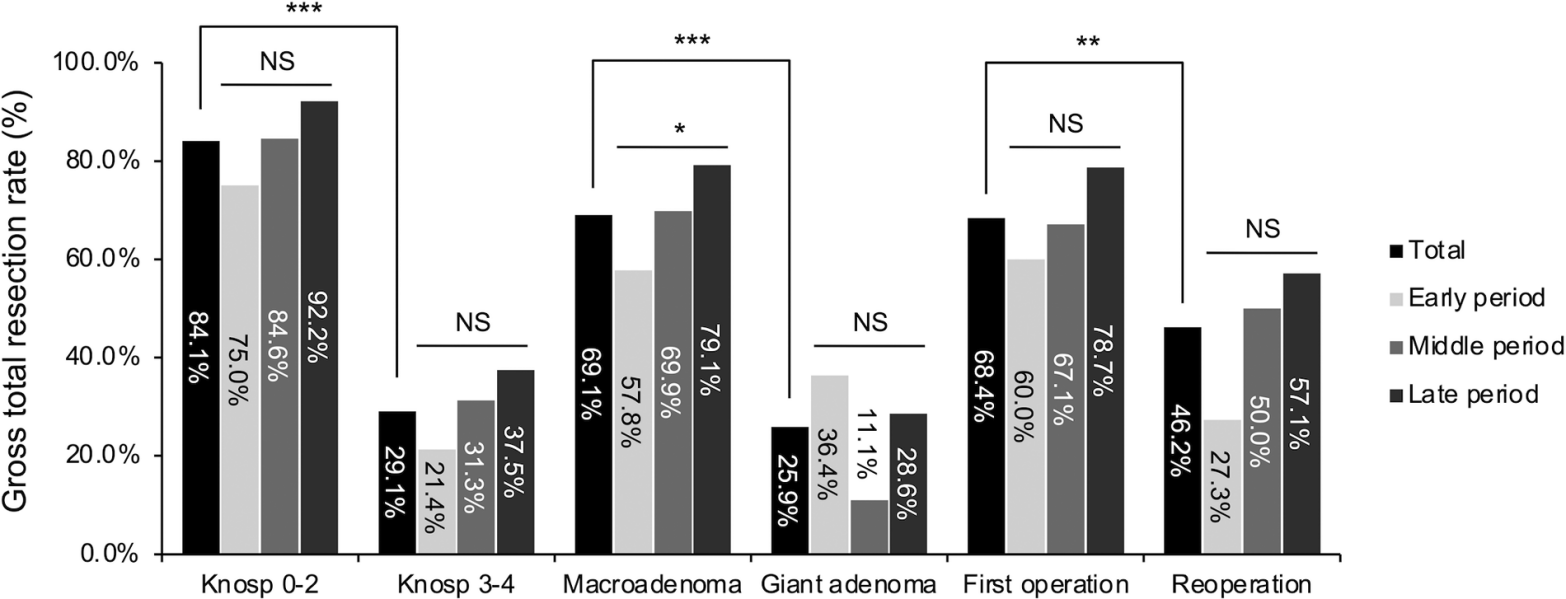
The gross total resection (GTR) rate by periods for pituitary adenomas with different surgical complexity. Comparison of total (black bars), early period (silver bars), middle period (light gray bars), and late period (dark gray bars). Data were compared by Chi-square test or Fisher's exact test when appropriate, * indicates *P* < 0.05, ** indicates *P* < 0.01, *** indicates *P* < 0.001, and NS indicates not significant (*P* > 0.05).

The rate of GTR for each subgroup was summarized in Figure 4. In comparison to their simple counterpart groups (84.1% for Knosp grade 0–2 adenomas, 69.1% for the macroadenomas, and 68.4% for patients undergoing first operation), the GTR rate was significantly lower for the complex groups (29.1% for Knosp grade 3–4 adenomas, *P* < 0.001; 25.9% for giant adenomas, *P* < 0.001; 46.2% for patients undergoing reoperation, *P* = 0.002). In terms of temporal trend, an increasing trend in GTR rate across the three periods was seen in each subgroup except the giant adenoma group. A statistically significant increase was only seen in the macroadenoma group (57.8% to 69.9% to 79.1%, *P* = 0.03). The results of multivariable logistic regression analysis showed that the surgical period was an independent factor for GTR only in simple groups [Knsop grade 0–2 adenoma: OR = 2.076 (95%CI 1.118–3.858, *P* = 0.021); macroadenoma: OR = 2.090 (95%CI 1.287–3.393, *P* = 0.003); first operation: OR = 1.809 (95%CI 1.104–2.966, *P* = 0.019)] but not in complex groups [Knosp grade 3–4 adenoma: OR = 1.509 (95%CI 0.810–2.810, *P* = 0.195); giant adenoma: OR = 0.688 (95%CI 0.206–2.291, *P* = 0.542); reoperation: OR = 1.890 (95%CI 0.740–4.828, *P* = 0.183)].

### Surgical outcomes in FPA

**Figure 5:**
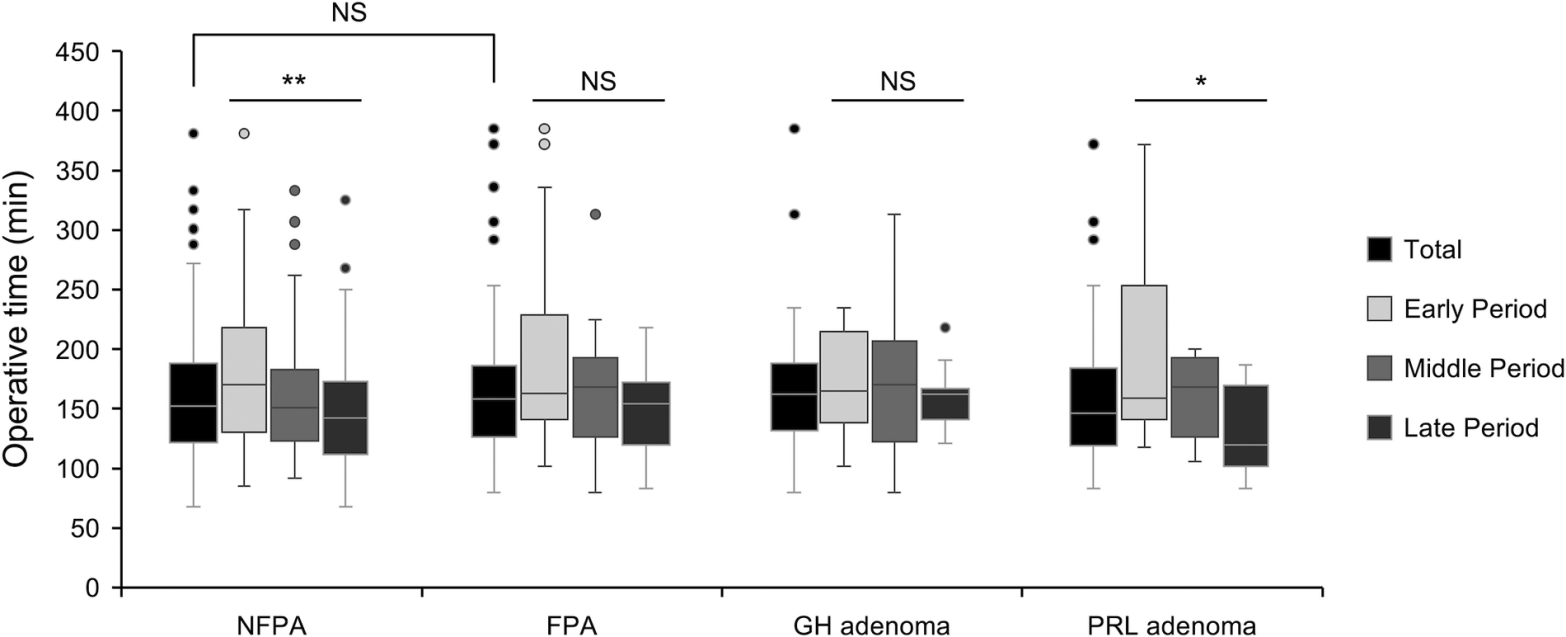
The operation time for non-functioning and functioning pituitary adenomas, GH-secreting adenomas, and PRL-secreting adenomas. Comparison of total (black bars), early period (silver bars), middle period (light gray bars), and late period (dark gray bars). Data were compared by the Kruskal-Wallis test, * indicates *P* < 0.05, ** indicates *P* < 0.01, and NS indicates not significant (*P* > 0.05). NFPA, non-functioning pituitary adenoma; FPA, functioning pituitary adenoma.

FPAs accounted for 33.3% of our series. The total operative time was 158 min (80–385 min, Figure 5) for FPAs and 152.0 min (68–381 min) for NFPAs, without a significant difference between the two groups. The operative time decreased significantly between periods in the NFPA group (171.0 to 150.0 to 142.0 min, *P* < 0.001), while no clear temporal trend in operative time was found in the FPA group (163.0 to 168.0 to 154.5 min, *P* = 0.076). For GH-secreting adenomas, the operative time showed no significant change across the three periods (*P* = 0.821). However, for PRL-secreting adenomas, the operative time in the late period was significantly lower than that in the early period (120 min vs. 159 min, *P* = 0.008).

46/91 (50.5%) of FPA achieved BC after surgery (Table 2). For GH-secreting adenoma, BC was achieved in 27 of 50 (54.0%) patients. The BC rate increased across periods, but insufficient numbers reached statistical significance (40.0% to 55.0% to 66.7%, *P* = 0.376). Only 44.7% of patients with PRL-secreting adenoma achieved BC. The BC rate of PRL-secreting adenomas varied obviously over time without a statistical difference (*P* = 0.273). The middle period had the highest remission rate at 71.4%. Furthermore, BC was achieved in one of 2 ACTH-secreting adenomas (50%) and one TSH-secreting adenoma (100%) after surgery.

### Complications

In our series, intraoperative CSF leakage occurred in 77 patients (28.2%), while postoperative CSF leakage occurred in 22 patients (8.1%). The incidence of intraoperative CSF leak increased slightly from 20.9% in the early period, 28.6% in the middle period to 35.2% in the late period (*P* = 0.101), while the incidence of postoperative CSF leak decreased slightly from 12.1% in the early period to 6.6% and finally to 5.5% in the late period (*P* = 0.216), though neither trend was statistically significant. Other complications included 27 transient diabetes insipidus (9.9%), 4 permanent diabetes insipidus (1.5%), 18 meningitis (6.6%), 7 epistaxes (2.6%), 2 internal carotid artery injuries (0.7%) and 1 death (0.4%).

## Discussion

The learning curve is particularly important in surgery, where new skills must be acquired safely and efficiently (12). Our present study reported the temporal trends in operative time, GTR, and BC rate of PAs with various degrees of surgical complexity. Unlike a dichotomization method used in most previous studies (8, 13–15), we divide the patients into three chronological groups, which would be helpful to describe the continuous temporal trend. Then, based on the tumor size (16–18), cavernous sinus invasion (17), and reoperation (8, 17), which were thought to be predictors of the extent of resection in previous studies and might be more indicative of surgical complexity, we divided patients into subgroups. The selection bias was taken into account, which may have been caused by our preference for treating relatively simple cases (first-time surgery, Knosp grade 0–2 adenoma, less suprasellar extension, better pneumatization of the sphenoid sinus) at the beginning of the transition from microscopic to endoscopic endonasal transsphenoidal surgery. Therefore, we first evaluated baseline characteristics between the three time periods and found no statistically significant differences in the distributions of tumor MD, Knosp grade, or previous pituitary surgery (*P* > 0.05). Moreover, these distributions were compared with those reported in previous studies focused on the learning curve in ETS for PA (7, 8, 14).

### Operative time

In our series, the median operative time was 165 min, significantly decreasing over time (*P* = 0.001). A similar downward trend in operative time was also found in previous studies (8, 13, 19). Additionally, we discovered another considerable reduction (30 min, 18.6%) in operative time in the final year after it had been relatively stable for several years (ranging from 151.5 to 163 min). This later improvement may be attributed more to proficiency with skills and the development of teamwork. It was previously thought that once the learning curve reached a plateau, performance would stabilize, and further repetitions would no longer contribute to improving performance (20). However, in the largest case study on the learning curve with 1,000 patients undergoing endoscopic skull base surgery, Younus et al. found that the tail end of the learning curve likely continues to slope upwards after several years or even decades (21). Similar learning curve characteristics were observed in uniportal video-assisted thoracoscopic lobectomy (22) and one-stage anterior urethroplasty (23).

The learning curve may be impacted by surgical complexity. However, only a few studies have looked at the learning curves for PA resection with various degrees of surgical complexity. The present study found that operative time decreased significantly in the simple group rather than their complex counterparts. This result revealed that complex operations would have a longer learning curve regarding operative time. Younus et al. also suggested that the most complex tasks have the longest learning curve (21). Surgery should be seen as a succession of skills. The surgical learning curve should synthesize the learning curve for each necessary skill. Complex surgeries usually require more skills than simple ones. For instance, exposing suprasellar or/and parasellar structures in addition to the sellar floor could be necessary when removing giant adenomas or Knosp grade 3–4 adenomas. Reoperation requires the surgeons to be able to identify the correct surgical path in disrupted anatomy and scars in addition to knowledge of normal anatomy. Therefore, it is reasonable to assume that mastering these challenging tasks will take more time and practice, resulting in a longer learning curve.

Surgical skill acquisition is a step-by-step process that progresses from simple to complex. In a cohort with a mix of cases of varying difficulty, reaching a plateau in operative time is more likely to reflect a balance between proficiency with simple skills and attempts at difficult skills. As new skills are acquired, the learning curve may continue to slope upward. Therefore, differentiating case complexity and then evaluating the learning curve, respectively, may be more helpful in determining the surgeon's learning level and allowing for more targeted practice, such as specific anatomical training or the addition of complex cases for manipulation.

### GTR rate

Total resection rates of PA have been reported to range from 58% to 88% (17, 18, 24–26). In our study, the GTR rate gradually increased over 7 years from 58.1% to 75%, showing a gradual and continuous learning process. The majority of learning curve studies demonstrated an increase in GTR rates over time. However, most examined patients by splitting into two chronological groups (17, 27), making it difficult to identify a long-term trend in the GTR rate. The study by Younus et al. provides a better picture of the long-term trend in GTR rates (7). They divided 600 pituitary tumor cases over 14 years into quartiles and showed that the overall GTR rate increased from 55% to 79% across quartiles. There was still a 12.9% increase between the last two quartiles. This result demonstrated that linear outcome increases occur even after 450 cases of the same surgical procedure. According to multivariable logistic regression analysis, the surgical period was an independent protective factor for GTR in the simple groups instead of the complex groups. The relationship between the surgical period and GTR showed the effect of surgical experience on improving the GTR rate in the simple groups. In the complex groups, no such effect was noticed. The requirement for a surgeon to master more techniques and skills for complex operations was one of the potential reasons. Another reason could be the limited number of cases.

### The learning curve for FPA

In contrast to NFPAs, FPAs must achieve BC and complete total resection demonstrated by postoperative MRI. Our study found that operative time decreased significantly across the three periods in NFPAs but not in FPAs. Similar results were observed in a prior study by Leach et al., in which they found a decrease in the duration of surgery between periods 1 and 2 for NFPAs, but not for functioning adenomas (28). We spend more time making sure the patients with FPAs achieve BC, which could neutralize the reduction in operative time brought on by proficiency.

Few articles have looked at the temporal trend in BC rate for different subtypes of FPA. Younus et al. have found that the remission rates increased for each subtype of hormone-producing adenoma without statistical significance (7). Our cohort of GH-secreting adenomas showed a similar increasing trend, whereas the PRL-secreting adenomas did not. The BC rate varied substantially across the three periods due to the high percentage of macroadenomas (94.7%) and Knosp grade 3–4 adenomas (47.4%) among our PRL-secreting adenomas, which were known to impact the surgical cure rate (29, 30). Meanwhile, depending on the tumor characteristics (tumor size or cavernous sinus invasion), the surgical goal for PRL-secreting adenomas may be maximal cytoreduction rather than aggressive GTR, which may also affect the surgical cure rate. Therefore, the BC rate trend for PRL-secreting adenomas is less likely to reflect the surgeon's surgical skill accurately.

### Complications

Regarding complications, perioperative CSF leakage was the most common variable measured in learning curve studies of ETS (15, 31, 32). The incidence of intraoperative CSF leakage was found to increase over time in our study. In a study of 142 PA cases undergoing transsphenoidal surgery, all three surgeons had more CSF leaks in the late group compared to the early group (33). Some studies also showed a higher incidence of intraoperative CSF leakage in the late group (19, 31), while in other studies (15, 21, 32), intraoperative CSF leakage significantly declined with increasing experience. The variation in results could be attributed to different surgeon characteristics. As our surgical technique and confidence in sellar reconstruction improved, a more aggressive resection strategy was more likely to be adopted to accomplish complete resection. Despite increased intraoperative CSF leakage, postoperative CSF leakage has steadily decreased. Previous studies have also found similar downward trends in postoperative CSF leakage (16, 28, 34, 35). It undoubtedly reflects the increasing proficiency with sellar reconstruction techniques.

### Limitations

One of the limitations of our study was the retrospective design. Second, we pooled data from three neurosurgeons on our team for better subgroup analysis. However, different learning stages of neurosurgeons will influence the results. Our three experienced neurosurgeons transitioned from microscopic to endoscopic transsphenoidal surgery during the same period, and the total number of endoscopic surgery cases each did during the study period was comparable. These may minimize the impact on results, although differences in surgeon characteristics and learning capacities should still be considered. Third, we divided patients into three periods without clinically significant change points, which was arbitrary, and the size of groups may hinder learning curve interpretation (12). Moreover, the criteria (Knosp grade, tumor size, and history of prior transsphenoidal surgery) we used to classify the complexity of surgery were limited. More variables and combinations will need to be used in future research. Finally, adding more cases may help draw more convincing conclusions in subgroup analysis.

## Conclusion

Our present study suggested that complex cases may have a longer learning curve regarding the operative time and GTR rate. Differentiating the complexity of cases and assessing the learning curve using multivariable may be useful in determining the learning level of surgeons and providing further targeted practice and training.

## Data Availability

The raw data supporting the conclusions of this article will be made available by the authors, without undue reservation.
